# Uncoupling of complex regulatory patterning during evolution of larval development in echinoderms

**DOI:** 10.1186/1741-7007-8-143

**Published:** 2010-11-30

**Authors:** Kristen A Yankura, Megan L Martik, Charlotte K Jennings, Veronica F Hinman

**Affiliations:** 1 Department of Biological Sciences, Carnegie Mellon University, Pittsburgh, PA 15213, USA

## Abstract

**Background:**

Conservation of orthologous regulatory gene expression domains, especially along the neuroectodermal anterior-posterior axis, in animals as disparate as flies and vertebrates suggests that common patterning mechanisms have been conserved since the base of Bilateria. The homology of axial patterning is far less clear for the many marine animals that undergo a radical transformation in body plan during metamorphosis. The embryos of these animals are microscopic, feeding within the plankton until they metamorphose into their adult forms.

**Results:**

We describe here the localization of 14 transcription factors within the ectoderm during early embryogenesis in *Patiria miniata*, a sea star with an indirectly developing planktonic bipinnaria larva. We find that the animal-vegetal axis of this very simple embryo is surprisingly well patterned. Furthermore, the patterning that we observe throughout the ectoderm generally corresponds to that of "head/anterior brain" patterning known for hemichordates and vertebrates, which share a common ancestor with the sea star. While we suggest here that aspects of head/anterior brain patterning are generally conserved, we show that another suite of genes involved in retinal determination is absent from the ectoderm of these echinoderms and instead operates within the mesoderm.

**Conclusions:**

Our findings therefore extend, for the first time, evidence of a conserved axial pattering to echinoderm embryos exhibiting maximal indirect development. The dissociation of head/anterior brain patterning from "retinal specification" in echinoderm blastulae might reflect modular changes to a developmental gene regulatory network within the ectoderm that facilitates the evolution of these microscopic larvae.

## Background

The astonishing diversity of animal forms, coupled with the complex life histories typical of many marine invertebrates, presents numerous challenges in inferring the ancestral character of members of the closely related phyla collectively known as the deuterostomes. Modern molecular phylogenies place four phyla within the monophyletic deuterostomes: Echinodermata and Hemichordata comprise a distinct clade called the Ambulacraria [1-3] that is a sister group to Chordata [4]. Xenoturbella is a recent out-group addition to the Ambulacraria [5].

Within the Ambulacraria, the free-swimming, bilaterally symmetric larvae of echinoderms, especially the bipinnaria larva of sea stars and the auricularia larva of sea cucumbers, share many similarities with the tornaria larva of indirectly developing hemichordates. These microscopic larvae have an apical concentration of serotonergic neurons [6] and one or two concentrations, or bands, of cilia used to feed and swim in the plankton [7,8]. Neurons lie beneath this ciliated epithelium and innervate the bands [9]. Similarities in larval form initially provided the basis for many of the hypotheses surrounding the evolutionary origins of the chordates and, in particular, the centralized nervous system. These hypotheses, in which a microscopic larval stage is assumed ancestral to the entire deuterostome clade, propose that a centralized nervous system evolved from an infolding of the larval ciliary bands [10-12].

Not all Ambulacrarians develop through a larval stage, however, and recent comparisons of regulatory gene expression have revealed that orthologs of many genes and signaling molecules involved in vertebrate neural patterning are expressed in spatially restricted domains along the anterior-posterior (AP) axis of the direct developing vermiform hemichordate juvenile *Saccoglossus kowalevskii *[13,14], a species that does not develop via a tornaria larva. This general correspondence in AP position of orthologs between the vertebrate and hemichordate nervous systems implies some homology in axial patterning. In addition, the broad ectodermal expression of these genes in hemichordates suggests that a diffuse panectodermal neural domain was the ancestral state of the deuterostome nervous system and that the centralization event occurred later within the lineage leading to the chordates. These findings and those of other researchers (reviewed in [15,16]) therefore negate the need to invoke a ciliated ancestor for deuterostomes as suggested by Garstang [10]. In addition, general homologies in axial expression patterns between vertebrates and direct developing protostomes have been observed as well [17], suggesting that common patterning mechanisms have been employed since the radiation of Bilaterians. Indirect developing larval forms have less obviously distinguished body axes and no strong homology of axial patterning and, as a result, appear derived and possibly secondarily simplified in comparison.

Here we examine the expression of regulatory gene orthologs that have known or suspected roles in patterning the axial neuroectoderm of many protostome and deuterostome embryos within the indirectly developing sea star, *Patiria miniata *(previously *Asterina miniata*), which forms a typical bipinnaria larva. We show that these genes are expressed in diffuse concentric ectodermal domains that pattern the early embryonic axis. Furthermore, we observe in the sea star a general correspondence of domains of orthologous gene expression to those found along the AP and dorsal-ventral (DV) axis of direct developing deuterostomes. In addition, we detect expression of retinal determining gene orthologs in the mesoderm of echinoderm larvae, but not within the ectoderm of gastrulating embryos. We discuss the role of this implied modularity of regulatory patterning during evolution.

## Results and discussion

### Isolation of sea star transcription factors

*P. miniata *orthologs of regulatory genes that have known or suspected roles in patterning the axial neuroectoderm of many protostome and deuterostome embryos were isolated using a candidate gene approach. Recombinants for the following seven genes that encode homeodomain proteins were obtained: *retinal homeobox *(*rx*), *optix-like homeobox 3 *(*six3*), *gastrulation and brain-specific homeobox *(*gbx*), *lim domain homeobox 2 *(*lhx2*) and *paired box homeobox 6 *(*pax6*), as well as members of the Nkx gene family, *nk2.1 *and *nk1*. We also identified partial sequences of *eyes absent *(*eya*), the ets family gene *pea3*, and two C2H2 zinc-finger genes, *zic *and *krupple-like factor 13 *(*klf13*). The following four winged-helix forkhead box genes were isolated: *foxq2*, *foxj1*, *foxd *and *foxg*. A complete list of orthologs, sequence lengths, and orthology of gene sequences is provided in Additional file 1.

### Animal-vegetal patterning of the sea star blastula ectoderm

**Figure 1:**
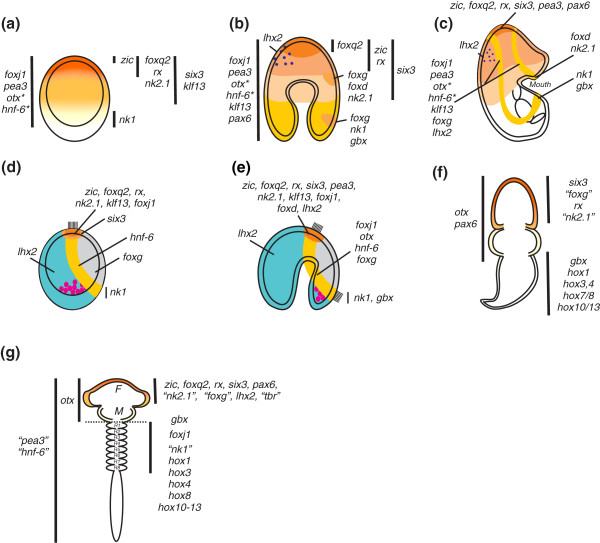
**Comparison of orthologous neuroectodermal gene expression domains among the deuterostomes**. **(A-E) **Indirectly developing echinoderms. **(F) **Directly developing hemichordate. **(G) **Generalized vertebrate. Sea stars (Figures 1A-1C) and sea urchins (Figures 1D and 1E) are viewed laterally; animal pole is up and oral side is right. Figures 1F and 1G are dorsal views; anterior is up. Genes are listed beside their cognate expression domains. Vertical bars in Figures 1A, 1B, 1F and 1G approximate domain boundaries. The orange to yellow gradient in Figures 1A, 1F and 1G reflects a general conservation of anterior (animal)-most axial patterning among the three phyla. **(A) **Nested, concentric expression domains pattern the animal-vegetal (AV) axis of blastulae; asterisks denote previously reported expression [18,19]. **(B) **Concentric domains of *zic*, *foxq2*, *rx *and *six3 *persist in gastrulae (orange to peach gradient); additional oral (for example, *foxg*, *foxd *and *gbx*; light orange) and aboral (for example, *lhx2*; purple) domains are evident. Genes (left) are broadly expressed. **(C) **Expression in larval animal pole domain (orange to peach) and/or ciliary bands (gold). **(D) **Sea urchin animal pole (orange and light orange), ciliary band (gold), aboral ectoderm (turquoise) and oral ectoderm (*foxg*; gray) are molecularly distinct territories in blastulae. **(E) **Expression is maintained in gastrulae animal pole (orange) and ciliary band (gold). **(D **and **E) **Pink circles represent skeletogenic mesoderm. See references [20,22,23,41-49]. **(F) **Orthologs expressed in hemichordate anterior, middle and posterior body segments show corresponding expression in the vertebrate forebrain, midbrain and hindbrain, respectively; data are summarized from Lowe *et al*. [13]. **(G) **Expression in generalized vertebrate centralized nervous system. F, forebrain; M, midbrain; R1-R8, rhombomeres of hindbrain. *zic *[50]; *pea3 *[51]; *hnf-6/onecut *[52]; and *tbr *[53]; *foxj1 *[54]; *hox *genes [55]. See references [24] and [26-32]. Echinoderm gene names (quotations) are substituted for simplicity in Figures 1F and 1G.

Sea star late blastulae have a morphologically distinct animal-vegetal (AV) axis that is first readily observed when elongation of cells at the vegetal pole results in a noticeable thickening of epithelium termed the *vegetal plate *[18]. During gastrulation, the vegetal plate invaginates to produce the mesoderm and endoderm of the larva, leaving the remaining animal epithelium as ciliated ectoderm [18]. At this stage, no obvious morphological differences in ectodermal cell type have been observed, but we nonetheless reveal here a remarkable complexity of regulatory states within the ectoderm (summarized in Figure 1).

**Figure 2:**
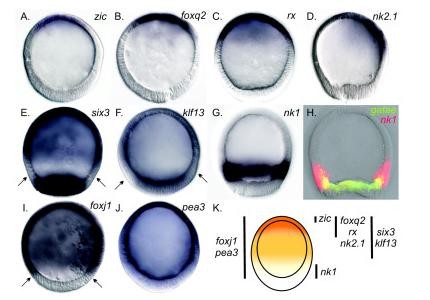
**Nested concentric expression domains pattern the axial ectoderm of sea star, *P. miniata*, blastulae**. Embryos are oriented with the animal pole up. **(A-G) **Whole mount *in situ *hybridization (*WMISH*). **(A) ***zic*, **(B) ***foxq2*, **(C) ***rx*, and **(D) ***nk2.1 *expression is restricted to the animal-most ectoderm. Transcripts of **(E) ***six3 *and **(F) ***klf13 *are detected in the ectoderm and in the vegetal plate endomesoderm. Arrows in **(E) **and **(F) **point to a clearing above the vegetal pole where no or few transcripts are detected. **(G) ***nk1 *transcripts are localized to a ring above the vegetal pole. **(H) **The boundary between the vegetal-most ectoderm (*nk1*, red) and the endoderm (*gatae*, green) as visualized by fluorescence *in situ *hybridization (*FISH*). Colocalization is in yellow. **(I **and **J) ***WMISH*. Transcripts of **(I) ***foxj1 *and **(J) ***pea3 *are detected throughout the entire ectoderm. *pea3 *is weakly detected in the vegetal plate endomesoderm. Arrows in Figure 1J point to the limits of *foxj1 *expression. **(K) **Schematic shows the patterns described above as five nested domains of expression along the AV axis. For simplicity, *nk2.1 *is grouped here with the concentric domains of *foxq2 *and *rx *expression. Gene names are listed next to their cognate expression domains. Vertical bars approximate the expression boundaries of associated genes. The color gradient spans the animal (orange) to vegetal (yellow) limits of the ectoderm.

Transcripts of sea star regulatory genes are localized throughout the animal ectoderm in overlapping concentric domains along the AV axis. Some of these transcripts, such as those of *zic*, *foxq2*, *rx *and *nk2.1*, are found only in the animal-most ectoderm (Figures 2A-2D). Of these genes, *zic *appears to be most closely localized to the animal pole, while expression of *foxq2 *and *rx *overlaps with *zic*, yet extends further. Transcripts of *six3 *and *klf13 *(Figures 2E and 2F) also are detected in the animal-most ectoderm of blastulae; however, they show a still broader distribution. Although there is no clear cell morphology that demarcates the boundary between the animal ectoderm and the vegetal plate endomesoderm, we observe a ring of *nk1 *expression above the vegetal plate (Figure 2G) that partially overlaps with endodermally localized *gatae *transcripts (Figure 2H). The *nk1 *expression domain therefore likely marks the vegetal-most ectoderm of the blastula. *foxj1 *and *pea3 *(Figures 2I and 2J) are expressed throughout the ectoderm.

Taken together, the spatial expression of these regulatory genes demonstrates that the ectoderm of the sea star blastula is patterned along the AV axis in at least five nested concentric domains (summarized in Figure 2K).

### Regulatory gene expression within the ectoderm of the ciliary bands and animal pole domain

**Figure 3:**
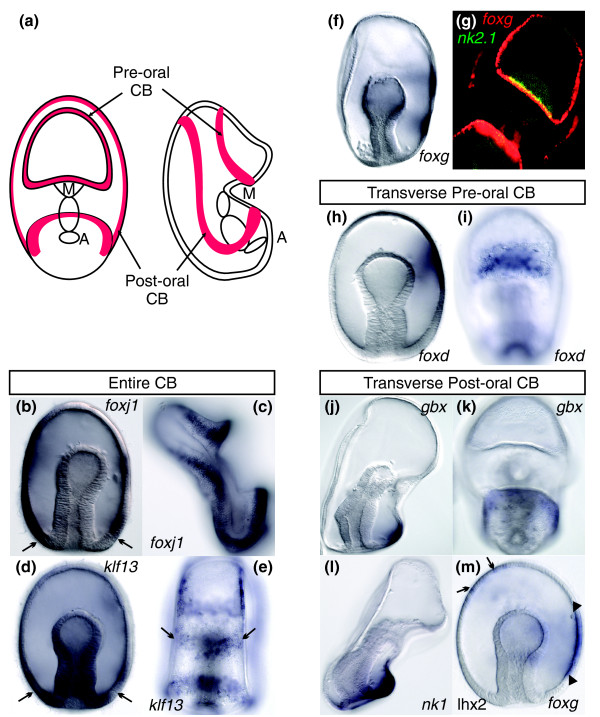
**Heterogeneous regulatory patterning of the larval ciliary bands as visualized by *WMISH***. **(A) **Schematic describes the position of the two larval ciliary bands (red) from oral (left) and lateral (right) views. A, anus; CB, ciliary band; M, mouth. **(B-F) ***WMISH*. Expression of **(B **and **C) ***foxj1 *and **(D **and **E) ***klf13 *is initially broad throughout **(B **and **D) **the ectoderm of gastrulae, then later is restricted to **(C **and **E) **the larval ciliary bands. Arrows in Figure 3B show the vegetal limits of *foxj1 *expression. Arrows in Figure 3D point to a clearing above the vegetal pole where transcripts of *klf13 *were detected. *klf13 *transcripts are additionally detected in an ectodermal territory near the mouth (arrows in Figure 3E). **(F) ***foxg *is first expressed within two ectodermal domains on the oral side of gastrulae. **(G) ***FISH *of *nk2.1 *(green) and ciliary band marker *foxg *(red) highlights *nk2.1 *expression in only the transverse preoral ciliary band. Colocalization is shown in yellow. **(H-M) ***WMISH. foxd *is expressed within a single domain in **(H) **the oral side ectoderm of gastrulae and **(I) **in the transverse, preoral larval ciliary band. *gbx *is expressed in one domain in **(J) **the oral side ectoderm in gastrulae and in **(K) **the transverse postoral larval ciliary band. **(L) ***nk1 *is expressed in the transverse postoral ciliary band in the larva. **(M) **A two-probe *WMISH *shows *lhx2 *expression in a spotted pattern in the aboral ectoderm (arrows, left) opposite of *foxg *expression (arrowheads, right). Embryos are oriented with the animal pole up and laterally, except in Figures 3E, 3G, 3I and 3K, which are oral views. In lateral views, the oral side is to the right.

During gastrulation, the ectoderm appears to undergo very little morphological change other than the coalescence of cilia within two bands: a preoral ciliary band that loops above the opening of the mouth and a postoral ciliary band that loops below it and around the aboral surface at the "back" of the embryo (Figure 3A) [7,18]. Transcripts of several genes that were distributed broadly throughout the ectoderm prior to gastrulation are later expressed within the ectoderm of the ciliary bands of the larva following gastrulation (for example, *foxj1 *and *klf13 *in Figures 3B-3E, *pea3 *as summarized in Figure 1 and as previously reported for *otx *and *hnf-6*/*onecut *expression [18,19]). At present, it is unclear if these patterns of expression reflect a migration of ectodermal cells to the sites of the future ciliary bands or if there is another patterning mechanism that restricts the earlier broad expression. Other transcripts are first detected at this stage within the ectoderm on the oral side of the gastrula and then later within the ciliary bands (for example, transcripts of *foxg*, *foxd *and *gbx*; Figures 3F and 3G, 3H and 3I, and 3J and 3K, respectively). A two-probe whole mount *in situ *hybridization (*WMISH*) of *foxg *and *lhx2*, a gene localized to the aboral ectoderm, further highlights the oral side restriction of *foxg *transcripts in the gastrula (Figure 3).

The expression patterns at this later stage also show that the regulatory state of the early larval ciliary bands is heterogeneous, for example, *nk2.1 *and *foxd *are expressed in part of the preoral ciliary band directly above the mouth (Figures 3G and 3I, respectively), while *gbx *and *nk1 *are localized to part of the postoral ciliary band below the mouth (Figures 3K and 3L, respectively). Therefore, while the regulatory state of the ciliary band ectoderm can be defined by a suite of transcription factors (that is, *klf13*, *foxj1*, *pea3*, *foxg*, *otx *and *hnf-6/onecut*), they are further subdivided into pre- and postoral regions on the basis of the localization of *foxd*, *nk2.1*, *nk1 *and *gbx*.

**Figure 4:**
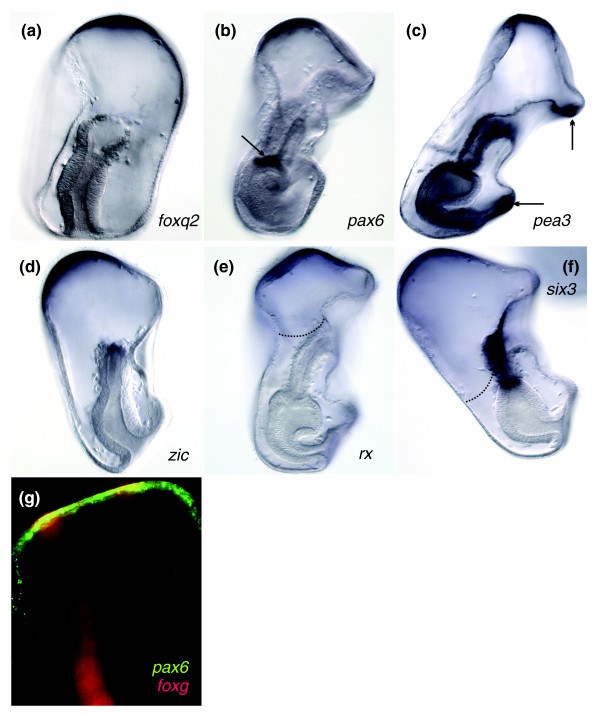
**Gene expression molecularly defines the animal pole domain in the sea star**. Embryos are shown laterally, with the animal pole up and oral side to the right. **(A-F) ***WMISH*. Expression of **(A) ***foxq2*, **(B) ***pax6*, **(C) ***pea3*, **(D) ***zic*, **(E) ***rx *and **(F) ***six3 *within the apical-most ectoderm defines the animal pole domain within late gastrulae (Figures 4A, 4D and 4F) and early larvae (Figures 4B, 4C and 4E). The vegetal limits of this domain are variable (see dotted lines in Figures 4E and 4F). Transcripts of *pea3 *additionally localize within the ectoderm of the larval ciliary bands (arrows in Figure 4C). *pax6 *expression in mesodermally derived coelom (arrow in Figure 4B). **(G) ***FISH *demonstrates that the ciliary bands, as marked by *foxg *(red), run through the ectoderm of the animal pole domain, as marked by *pax6 *(green). Colocalization is shown in yellow.

Other transcripts that we detected within the ectoderm of the blastula remained within the animal ectoderm as gastrulation proceeded in what we define here as the animal pole domain. Unlike sea urchins, the sea star, *P. miniata*, does not appear to have a morphologically distinct animal pole domain at this stage. Transcripts of *foxq2*, *pax6 *and *pea3 *(Figures 4A-4C) tightly localize to the animal pole ectoderm, although their vegetal boundaries do not exactly coincide. Transcripts of *zic*, *rx *and *six3 *are expressed within the animal pole domain as well, but even more vegetally throughout the animal ectoderm (Figures 4D-4F). The vegetal boundary of the animal pole domain therefore is not clearly defined by regulatory gene expression. The preoral and postoral ciliary bands run through the sea star animal pole domain as demonstrated by a two-probe fluorescence *in situ *hybridization (*FISH*) using the ciliary band marker, *foxg*, and the animal pole domain gene, *pax6 *(Figure 4G). Thus, despite its lack of morphological regionalization, the animal pole has a distinct regulatory state, as defined by *foxq2*, *pax6*, *pea3*, *zic*, *rx *and *six3 *expression, suggesting that it is a unique territory within the sea star. It is not yet clear whether these genes remain expressed in all cells of the sea star animal pole domain during later stages of larval development or if expression becomes refined to only subsets of cells within this domain.

### Comparisons of ectodermal patterning between sea urchin and sea star embryos

At first inspection, the expression patterns of many genes appear markedly different in the earlier blastula stages of sea urchin and sea star embryos. The later restrictions within the animal pole or ciliary bands are, with some exceptions, more similar (Figure 1). We suggest that the sea urchin embryo may simply undergo a relatively more rapid specification of these territories, with an associated loss of intermediate domains that we observe in the sea star. Indeed, a careful examination of expression patterns in sea urchin has recently shown that the apical plate in sea urchin consists of at least two regulatory domains: an inner animal pole domain flanked by a ring of *six3 *expression [20]. These two domains in the sea urchin hatched blastula may therefore represent a more apically compressed version of the nested, concentric regulatory domains found in the sea star blastula.

Some of the patterning differences between sea urchin and sea star ectoderm also seem to account for the differences observed in the localization of the pan-neuronal marker, synaptotagmin-B [21]. For example, similar to the patterns of gene expression that we describe here, synaptotagmin-B is detected broadly throughout the ectoderm of the sea star gastrula, but in the larva it is found primarily in neurons associated with the ciliary bands and animal pole [9]. In the sea urchin, however, synaptotagmin-B is already localized to the animal pole domain and the presumptive ciliary band by the gastrula stage [9].

Although expression of many of the genes within the ciliary bands of the sea star appears conserved in the sea urchin, *nk2.1 *and *foxd *show clear differences in expression that may be associated with the evolutionary transition from a double looping of the ciliary band around the body of the sea star bipinnaria and hemichordate tornaria to a single looping of the ciliary band observed in sea urchins. This single ciliary band in the sea urchin develops at the junction between oral and aboral ectoderm. *nk2.1 *and *foxd *are expressed in part of the preoral ciliary band of the oral hood of the sea star, while sea urchin orthologs of these are found in the animal plate ectoderm (compare Figures 1C-1E). Interestingly, both of these genes in sea urchin appear enriched on the oral side of the embryos [22,23]. Thus, we speculate that the preoral ciliary band may have been compressed into the oral-side animal plate territory in sea urchins and that this region within the sea urchin may therefore constitute a different territory than the remaining animal plate.

### Conservation of anterior (animal)-most regulatory patterning with other deuterostomes

Comparisons of the regulatory gene expression patterns that we observed in these indirectly developing sea star embryos with those known in directly developing bilaterians illuminate additional surprising patterns of conservation. We observe a general mapping of gene expression patterns along body axes (compare Figures 1A-1C with Figures 1F-1G). For instance, in the sea star, *foxq2*, *rx*, *pax6 *and *six3 *orthologs are apically expressed within the ectoderm. *foxq2 *expression in the amphioxus, a basal chordate, is restricted to the anterior-most end of the embryo [24]. Orthologs of *rx *and *pax6 *are expressed in the anterior-most neuroectoderm in the hemichordate *Saccoglossus *[13], and they also pattern the anteriorly localized eye primordium in vertebrates [25,26]. The *Drosophila rx *ortholog is required for brain development [27]. Orthologs of *six3 *and *otx *are expressed in anterior neuroectoderm in members of all three deuterostome phyla [13,28,29]. The most vegetal ectoderm in sea stars is characterized by the presence of *nk1 *and *gbx *transcripts. In vertebrates, a *gbx *ortholog establishes the midbrain-hindbrain boundary [30]. The zebrafish ortholog of *nk1*, *sax2*, is expressed within the midbrain-hindbrain boundary as well, although its expression is not exclusive to this territory [31]. Expression of *nk1 *and *gbx *in sea stars, and possibly sea urchins, marks the vegetal (posterior)-most ectoderm.

There is some evidence of additional conservation between the DV and oral-aboral axes as well. The mouse ortholog of *nk2.1 *(*nkx2.1*) is involved in the formation of motor neurons in the ventral telencephalon [32]. *Saccoglossus nk2.1 *orthologs also show a ventral bias in expression [13]. Furthermore, *foxg *plays a role in ventral forebrain development, while *lhx2 *specifies dorsal telencephalic fates [32]. We similarly show that expression of sea star orthologs of *foxg *and *nk2.1 *is restricted to the oral (ventral) ectoderm, while *lhx2 *orthologs are expressed within the aboral (dorsal) ectoderm (Figure 1B).

While in these comparisons we do not intend to convey a tight homology in gene expression patterns across deuterostome phyla, we predict that similarities in the overall patterning are an ancestral innovation and perhaps evidence of maintenance of some elements of a developmental gene regulatory network (GRN) inherited from a common ancestor. Conservation, however, is not maintained for orthologs of genes expressed within regions posterior to the midbrain-hindbrain boundary in chordates and hemichordates as *nk1 *marks the vegetal-most ectoderm. Also, the overlapping expression of *hox *gene orthologs needed to pattern the posterior of many embryos are found only later in echinoderm development within the mesoderm of the rudiment [33].

### Separation of "retinal" from "anterior neural" regulatory patterning

Vertebrate orthologs of transcription factors such as *pax6*, *six3 *and *rx *play known roles in pattering and specifying anterior vertebrate sensory systems, most notably the eyes [25,26,34]. Furthermore, orthologs of *pax6*, the *six *gene family members and *eya *operate in a similar gene network for retinal determination in both vertebrates and *Drosophila *(as reviewed in [35]).

Having established that orthologs of many regulatory genes involved in anterior neural specification are also expressed within the anterior ectoderm of sea star embryos, we sought to determine if orthologs of transcription factors involved within the retinal determination network are also expressed within echinoderm embryos.

**Figure 5:**
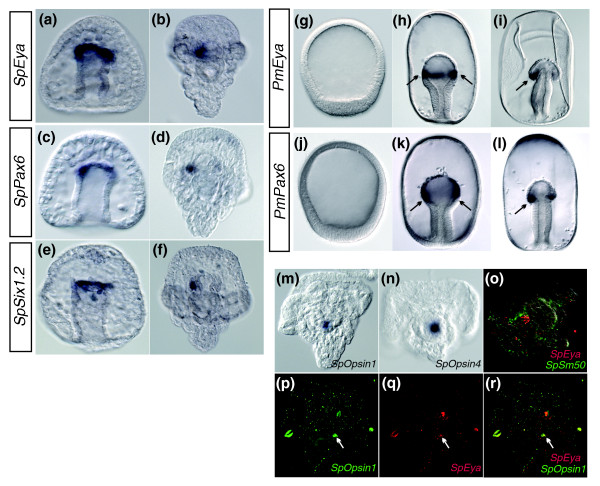
**Retinal determination orthologs are expressed within sea urchin, (*Strongylocentrotus purpuratus, Sp*), and sea star mesoderm**. **(A-N) ***WMISH*. **(A **and **B) ***SpEya*, **(C **and **D) ***SpPax6 *and **(E **and **F) ***SpSix1.2 *are expressed at the tip of the archenteron in gastrulae (Figures 5A, 5C and 5E) and in a mesodermally derived coelom in early pluteus larvae (Figures 5B, 5D and 5F). *PmEya *(Figures 5G-5I) and *PmPax6 *(Figures 5J-5L) expression is first detected in the mesoderm of the archenteron bulb in midgastrulae (Figures 5H and 5K; arrows) and then more prominently in a mesodermally derived coelom in late gastrulae (Figures 5I and 5L; arrow). *PmPax6 *transcripts are also found broadly throughout the ectoderm (Figure 5K), with more pronounced expression in the apical ectoderm (Figure 5L). *SpOpsin1 *(Figure 5M) and *SpOpsin4 *(Figure 5N) are expressed in 1-week-old larvae. **(O-R) ***FISH *in 1-week-old larvae. *SpEya *(Figure 5O) is shown relative to the skeletal marker *SpSm50*, which was used to orient the embryo. Figures 5P-5R show transcripts of *SpOpsin1 *(green) colocalizing with those of *SpEya *(red). Colocalization is shown in yellow. Arrows in Figures 5P, 5Q and 5R point to expression. Additional areas of expression within the 1-week-old larvae may be a result of nonspecific staining.

We have already shown that the sea star *pax6 *ortholog is expressed within the animal pole domain (Figure 4B), although it is not expressed within the ectoderm of the sea urchin embryo (Figure 5). Transcripts of both *pax6 *and *eya *in both sea urchins and sea stars, however, are detected in the mesoderm of midgastrulae and then more prominently in one mesodermally derived coelom in late gastrulae (Figures 5A-5D and Figures 5G-5L). While we were unable to obtain a *six1.2 *ortholog from the sea star, this gene is expressed also within the mesodermal coelom in the sea urchin (Figures 5E and 5F).

In the sea urchin, expression of two members of the light-sensing rhodopsin family of G-coupled protein receptors, *opsin1 *and *opsin4*, has been shown as early as 1 week [36]. We were unable to obtain sea star opsin sequences; however, we confirmed the expression of *opsin1 *and *opsin4 *1-week-old sea urchin larvae (Figures 5M and 5N). The morphology of the late larval sea urchin embryos makes it difficult to decipher the precise location of these transcripts within the embryo. We therefore sought to determine if *opsin*s collocalize with *eya*, which we show is expressed likely within one or both coeloms (depending on developmental timing) in 1-week-old larvae (Figure 5O). Using a two-probe *FISH*, we observe that transcripts of *opsin1 *colocalized with those of *eya *in 1-week-old sea urchin larvae (Figures 5P-5R). Expression of retinal determination orthologs within the mesoderm of gastrulae and larvae allow for the possibility that these genes operate within a common GRN.

The tightly coupled GRNs for anterior neural and visual sensory structures that are found in vertebrates and also in invertebrates, such as *Drosophila*, therefore are spatially separated in echinoderms. The presence of gene transcripts of *pax6*, *rx *and *six3*, but not, for example, *eya*, within the animal ectoderm of sea star bipinnaria larva may indicate a partial retention of an ancestral retinal determination network that once operated within this embryonic territory. This might also explain the absence of apically localized rhabdomeric eyespots, which are characteristic of the indirectly developing tornaria larvae of some hemichordates but were likely lost in the echinoderm lineage [16].

## Conclusions

### Inferring ancestral states

The detailed expression analyses reported here support the hypothesis that indirectly developing planktonic echinoderm embryos likely utilize ancient regulatory mechanisms for various anterior neuroectodermal and/or sensory developmental processes that are potentially conserved throughout the Bilateria. Compared to vertebrates and well-studied protostome model organisms such as *Drosophila*, however, echinoderm embryos separate the deployment of these subcircuits in space and/or time. Thus, echinoderm embryos may have conserved sets of genetic regulatory relationships for "head/anterior brain" within the ectoderm of the early blastula and others for "retinal determination" within the mesodermal coelom of the gastrula.

Much of the difficulty in inferring the ancestral state of the deuterostomes and the mysteries of the origin of the phylum to which we belong arises from the complex life histories found within extant lineages [37]. Given the conservation of complex sensory and AP patterning between protostomes such as *Drosophila *and vertebrates, the parsimonious explanation is that ancestral developmental regulatory interactions, perhaps even entire GRN subcircuits, have been uncoupled along the lineage of echinoderms, possibly coincident with a simplification in early development. However, until a greater breath of taxa have resolved GRNs, we cannot know the flexibility with which modular subcircuits can be deployed during evolution of alternative body plans or if intercalation of GRN subcircuits occurs independently or coincident with an increase in complexity.

## Methods

### Sea star and sea urchin embryo culture and characterization of gene expression

*P. miniata *embryos, previously named *Asterina miniata*, and *Strongylocentrotus purpuratus *embryos were cultured as described previously [18,38]. Partial gene sequences were obtained via screening a 3-day-old (late-gastrula stage) *P. miniata *arrayed cDNA library using *S. purpuratus *sequence-specific probes and low stringency conditions as previously described [39]. Whole mount *in situ *hybridization (*WMISH*) was performed as described previously [18].

### Two-color *FISH*

*WMISH *was performed essentially as described by Hinman *et al*. [18], with modifications to detect riboprobes using fluorescence as described by Denkers *et al*. [40]. In brief, both digoxygenin (DIG) and 2,4-dinitrophenol (DNP) labeled riboprobes were used. Hybridized probes were detected using anti-DIG antibody (1:2,000; Roche: Indianapolis, IN, USA) and anti-DNP antibody (1:1,000; PerkinElmer: Chicago, IL, USA), both conjugated to horseradish peroxidase, and the Tyramide Signal Amplification (TSA) Plus Fluorescence Systems Kit (PerkinElmer). A CyIII- or fluorescein-labeled tyramide was deposited near the *in situ *riboprobe in a reaction catalyzed by horseradish peroxidase, allowing for fluorescence detection of DIG- and DNP-labeled riboprobes.

## Competing interests

The authors declare that they have no competing interests.

## Authors' contributions

VFH and KAY conceived of the study, participated in its design and drafted the manuscript. KAY cloned sea star orthologs and performed *WMISH *and *FISH*. CKJ cloned sea urchin orthologs and performed WMISH. MLM cloned sea star orthologs and performed *WMISH *and *FISH*. All authors read and approved the final manuscript.

## Supplementary Material

Additional file 1 **Table 1**. List of sea star, *P. miniata*, orthologs and orthology of gene sequences. Click here for file
