# Supplementary material for: Uncoupling of complex regulatory patterning during evolution of larval development in echinoderms
Source: BMC Biol. 2010 Nov 30;8:143. doi: 10.1186/1741-7007-8-143 (PMC3002323; doi:10.1186/1741-7007-8-143)
Supplement: Additional file 1 — Table 1. List of sea star, P. miniata, orthologs and orthology of gene sequences. [file 1741-7007-8-143-S1.doc]

| **Ortholog** | **Length (nt)** | **Accession Number** | **Closest homologs** |
| --- | --- | --- | --- |
|  |  |  |  |
| *rx* | 1258 | HQ128718 | retinal homeobox (*S*. *kowalevskii*) |
|  |  |  | retinal homeobox 2A (*X*. *laevis*) |
|  |  |  |  |
| *six3* | 1301 | HQ148089 | similar to six3 (*S*. *purpuratus*) |
|  |  |  | sine oculis-related homeobox 3 homolog (*M*. *musculus* ) |
|  |  |  |  |
| *gbx* | 2684 | HQ128707 | gastrulation brain homeobox (*B*. *floridae*) |
|  |  |  | unplugged (*D*. *melanogaster*) |
| *lhx2* | 914 | HQ128716 | lhx2 (*S*. *purpuratus*) |
|  |  |  | lim homeobox 2/9 protein (S. *kowalevskii*) |
|  |  |  | lhx2 (*M*. *musculus*) |
|  |  |  |  |
| *pax6* | 1243 | HQ128717 | pax-6 (*P*. *lividus*) |
|  |  |  |  |
| *nk2.1* | 904 | HQ128710 | homeodomain protein nk2.1 (*S*. *purpuratus*) |
|  |  |  | nkx2-1 (*S*. *kowalevskii*) |
|  |  |  | nk2 homeobox 1 (*M*. *musculus*) |
|  |  |  |  |
| *nk1* | 520 | HQ128709 | homeobox transcription factor nk1 (*S*. *purpuratus*) |
|  |  |  |  |
| *eya* | 1141 | HQ128713 | eya1 protein (*D. rerio*) |
|  |  |  | eya1 protein (*M. musculus*) |
|  |  |  |  |
| *pea3* | 937 | HQ128711 | ets-related protein (*S*. *kowalevskii*) |
|  |  |  | polyoma enhancer protein 3 (*P*. *lividus*) |
|  |  |  |  |
| *zic* | 1541 | HQ148090 | zinc finger protein Ap-Zic (*A*. *pectinifera*) |
|  |  |  |  |
| *klf13* | 1036 | HQ128708 | krueppel-like factor 13 (*D*. *rerio*) |
|  |  |  |  |
| *foxq2* | 1943 | HQ148091 | forkhead transcription factor Q2 (*S*. *purpuratus*) |
|  |  |  | AmphiFoxq2 (*B*. *floridae*) |
|  |  |  |  |
| *foxj1* | 1141 | HQ128715 | forkhead transcription factor J1 (*S*. *purpuratus*) |
|  |  |  | forkhead box J protein (*S*. *kowalevskii*) |
|  |  |  |  |
| *foxd* | 1178 | HQ128712 | forkhead transcription factor D (*S*. *purpuratus*) |
|  |  |  | forkhead box D3 *X*. *laevis* |
|  |  |  |  |
| *foxg* | 1215 | HQ128714 | forkhead transcription factor G (*S*. *purpuratus*) |
|  |  |  | brain factor 1 (*S*. *kowalevskii*) |
|  |  |  | forkhead box G1 (*M*. *musculus*) |

Table 1. List of sea star, *P*. *miniata*, orthologs and orthology of gene sequences. Transcription factors isolated from a three-day-old *P*. *miniata* cDNA library are listed (Column 1). Sequence length in number of nucleotides (nt) is provided (Column 2). GenBank accession numbers for each sea star ortholog is given (Column 3). Orthology of gene sequences was determined using BLASTx (Column 4).
